# Supplementary material for: Proteomics and Microbiota Conjoint Analysis in the Nasal Mucus: Revelation of Differences in Immunological Function in Manis javanica and Manis pentadactyla
Source: Animals (Basel). 2024 Sep 14;14(18):2683. doi: 10.3390/ani14182683 (PMC11428827; doi:10.3390/ani14182683)
Supplement: Supplementary file 1 [file animals-14-02683-s001.zip › animals-3098916-supplementary.pdf]

Supplementary Table S1. The detailed information and TMT reagent labelled of pangolins

**Names**	**Species**	**Gender**	**Sampling location**	**Isotope labelling**
M-XWZ	*M. javanica*	Male	Shenzhen City	126
M-XTY	*M. javanica*	Female	Shenzhen City	127N
M-D1	*M. javanica*	Female	Shenzhen City	127C
M-D3	*M. javanica*	Male	Shenzhen City	128N
M-S3	*M. javanica*	Male	Shenzhen City	128C
Z-Z4	*M. pentadactyla*	Female	Guangzhou City	129N
Z-Z6	*M. pentadactyla*	Female	Guangzhou City	129C
Z-Z12	*M. pentadactyla*	Female	Guangzhou City	130N
Z-S1	*M. pentadactyla*	Male	Shenzhen City	130C
Z-S2	*M. pentadactyla*	Male	Shenzhen City	130

Supplementary Table S2. Sequences of primers employed for gene amplification and vector construction.

Names	Primer sequence (5’-3’)
16S-amplification of bacteria	FW: AGAGTTTGATCCTGGCTCAG
	RW: GGTTACCTTGTTACGACTT
C5a-amplification of *M. javanica*	FW: ATGCTGAATAAGAAAATAGA
	RW: ACATGAAATGTTTAGAGTAC
C5a-pET-32a of *M. javanica*	FW: CGGAATTCATGCTGAATAAGAAAATAGA
	RW: CCAAGCTTACATGAAATGTTTAGAGTAC
C5a-amplification of *M. pentadactyla*	FW: ATGCTGAATAAGAAAATAGA
	RW: CCTTCCCAACTGGATTTTTT
C5a-pET-32a of *M. pentadactyla*	FW: CGGAATTCATGCTGAATAAGAAAATAGA
	RW: CCAAGCTTCCTTCCCAACTGGATTTTTT

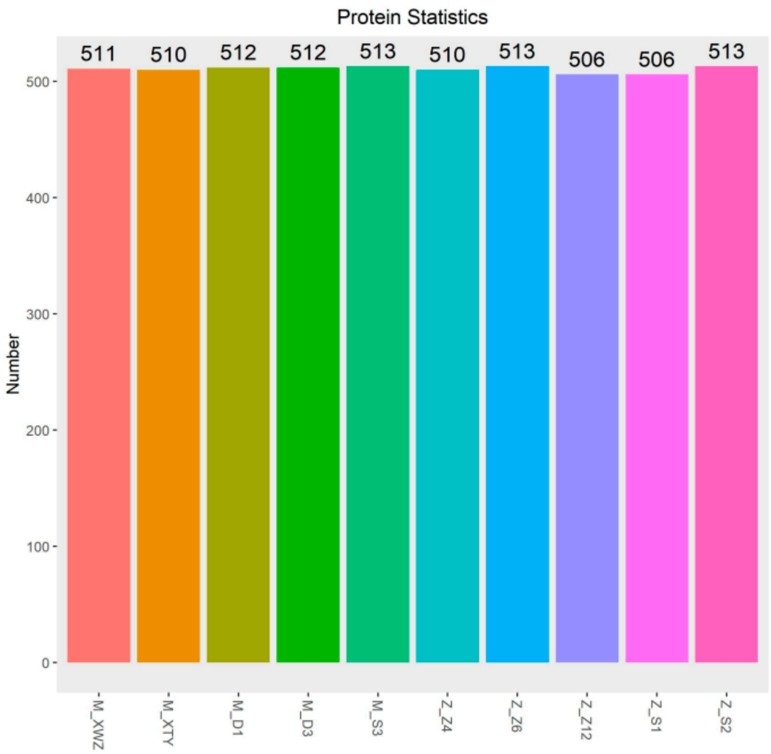

Supplementary Figure S1. The number of identified proteins in each samples.

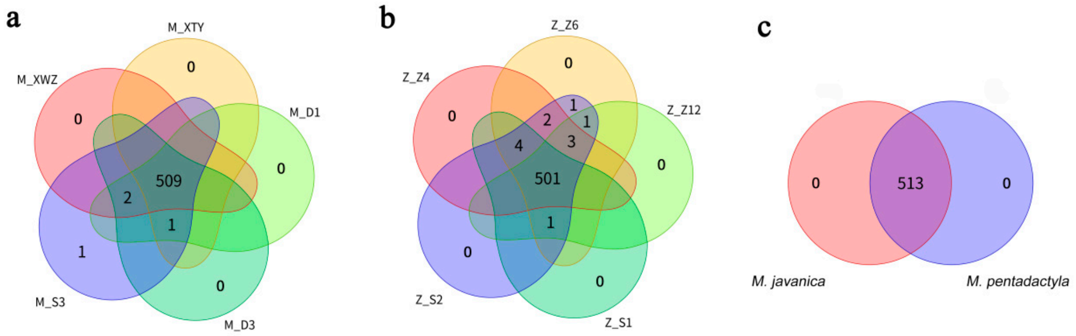

Supplementary Figure S2. Venn diagrams of different protein types in the nasal mucus of *M. javanica* **(a)**, *M. pentadactyla* **(b)** and between *M. javanica* and *M. pentadactyla* **(c)**.

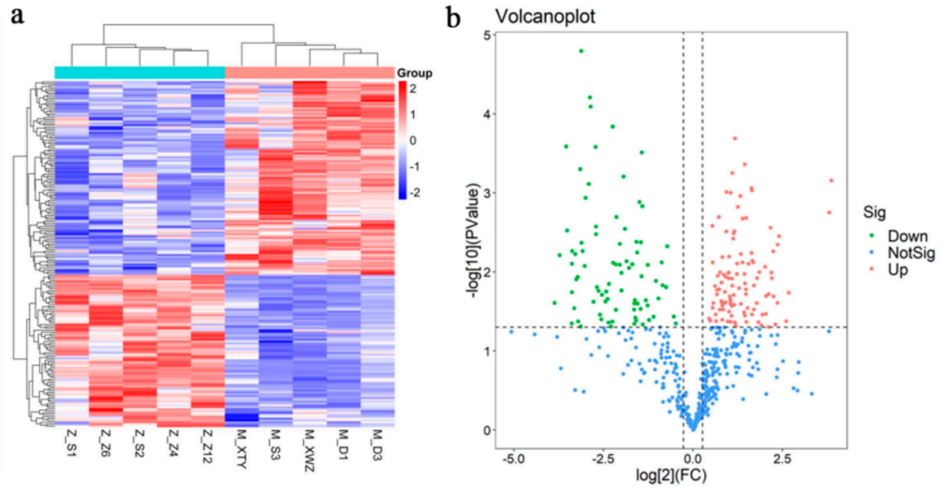

Supplementary Figure S3. Volcano plots **(a)** and hierarchical cluster analysis **(b)** of differentially expressed proteins in the nasal mucus of *M. javanica* and *M. pentadactyla.*

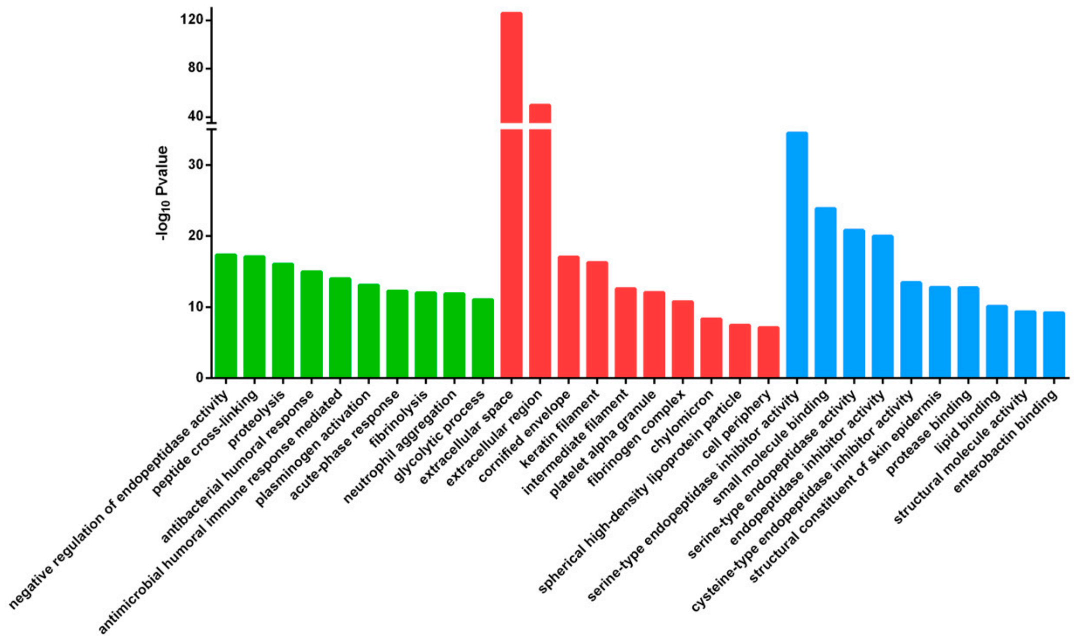

Supplementary Figure S4. The enriched GO terms (top 10) concluding the biological process (BP, green), cellular component (CC, red), and molecular function (MF, blue) of abundant proteins in the nasal mucus of *M. javanica* and *M. pentadactyla*.

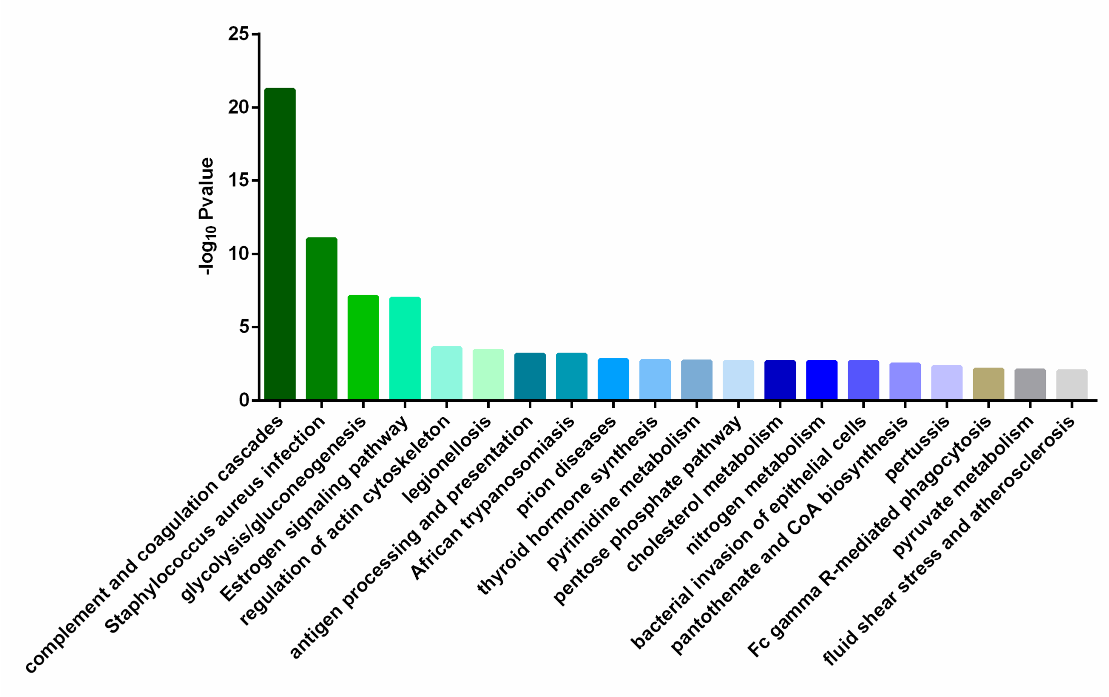

Supplementary Figure S5. KEGG pathway classification and functional enrichment of abundant proteins in the nasal mucus of *M. javanica* and *M. pentadactyla* (top 20 KEGG pathways).

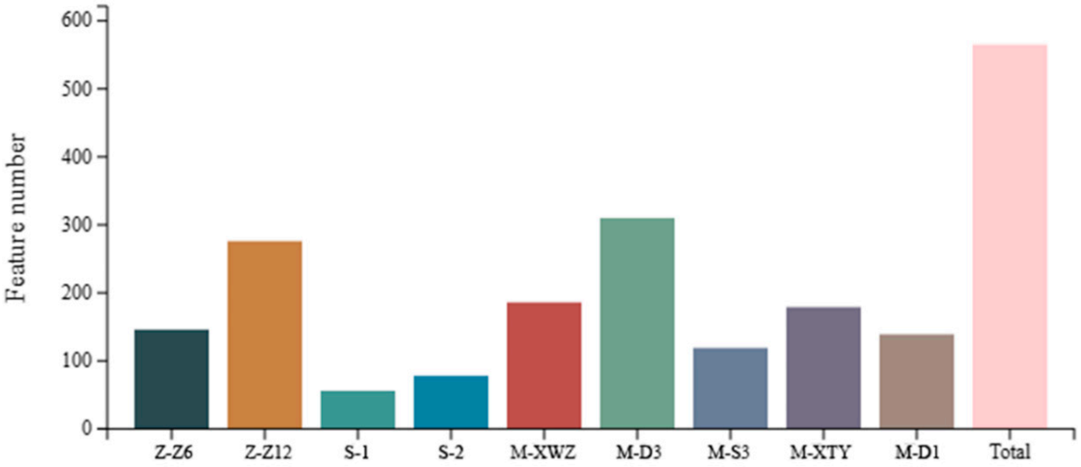

Supplementary Figure S6. The number of identified features in each samples.
